# Preoperative Nutritional Therapy Reduces the Risk of Anastomotic Leakage in Patients with Crohn's Disease Requiring Resections

**DOI:** 10.1155/2016/5017856

**Published:** 2015-12-27

**Authors:** Zhen Guo, Dong Guo, Jianfeng Gong, Weiming Zhu, Lugen Zuo, Jing Sun, Ning Li, Jieshou Li

**Affiliations:** Department of General Surgery, Jinling Hospital, Medical School of Nanjing University, No. 305 East Zhongshan Road, Nanjing 210002, China

## Abstract

*Background. *The rate of anastomotic leakage is high in surgeries for Crohn's disease, and therefore a temporary diverting stoma is often needed. We conducted this study to investigate whether preoperative nutritional therapy could reduce the risk of anastomotic leakage while decreasing the frequency of temporary stoma formation. *Methods.* This was a retrospective study. Patients requiring bowel resections due to Crohn's disease were reviewed. The rate of anastomotic leakage and temporary diverting stoma was compared between patients who received preoperative nutritional therapy and those on a normal diet before surgery. Possible predictive factors for anastomotic leakage were also analyzed. *Results.* One hundred and fourteen patients undergoing 123 surgeries were included. Patients in nutritional therapy (NT) group had a significantly lower level of C-reactive protein on the day before surgery. Patients in NT group suffered less anastomotic leakage (2.3% versus 17.9%, *P* = 0.023) and less temporary diverting stoma (22.8% versus 40.9%, *P* = 0.036). Serum albumin of the day before surgery ≤35 g/L and preoperative nutritional therapy were identified as factors which independently affected the rate of anastomotic leakage. *Conclusion.* Preoperative nutritional therapy reduced the risk of anastomotic leakage and the frequency of temporary diverting stoma formation in patients with Crohn's disease requiring resections.

## 1. Introduction

Crohn's disease (CD) is a chronic inflammatory gastrointestinal disease characterized by transmural inflammation which can cause microperforations, strictures, and fistulae. These complications are difficult to treat medically, and an estimated 80% of patients with CD will require at least one surgery such as resection or colectomies over their lifetime [1]. Unfortunately, in CD the risk of anastomotic complications (mainly anastomotic leakage) is higher than other benign intestinal diseases because of malnutrition, severe intestinal inflammation, immunosuppressive medication prior to surgery, duration of symptoms leading to surgery, and the complexity of the surgery [2–4]. Intestinal anastomotic complications are associated with increased mortality and morbidity. Therefore, a temporary stoma followed by delayed anastomosis has often been performed to minimize the risk of anastomotic complications in CD patients. Up to 39–51% of patients require this two-stage procedure [4–7].

However, stomas are not ideal and are preferably avoided. As emergency surgery is seldom necessary in CD, most patients can be best prepared for the surgery through preoperative management [8]. The nutritional therapy is an essential component for CD treatment, and it has become a preferred therapeutic strategy for the treatment of CD in some medical centers [9, 10]. Nutrition can not only improve nutritional status, but also reduce intestinal inflammation and induce mucosal healing, which can correct some risk factors of intra-abdominal septic complications. Previous studies have reported that preoperative management including nutritional therapy can reduce postoperative septic complications of CD [11, 12]. But whether preoperative nutritional therapy can reduce anastomotic leakage and temporary diverting stoma formation as well has not been well studied.

The purpose of this retrospective study was to investigate whether preoperative nutritional therapy could reduce the rate of anastomotic leakage without increasing the temporary stoma formation.

## 2. Materials and Methods

Patients treated for CD at inflammatory bowel disease (IBD) treatment center of our hospital have been prospectively registered in a database. Patients' baseline characteristics such as gender and age, medical treatment, indication for surgery, details of surgical procedures, complications, and details of follow-up were recorded by the gastroenterologists and surgeons.

### 2.1. Patients

All consecutive adult patients (age from 18 to 75) who underwent elective intestinal resection for ileal or ileocolonic or colonic CD due to intestinal strictures, fistula, or abdominal abscess were reviewed. CD diagnosis was confirmed by radiographic, endoscopic, histologic, and clinical findings. Data of age at operation, gender, body mass index (BMI), location and behavior of disease, duration of disease, levels of serum albumin and C-reactive protein (CRP), previous intestinal resection, preoperative medical treatment, indications for surgery, preoperative management, indications for temporary diverting stoma, surgical procedure, and anastomotic complications were obtained. Preoperative nutritional therapy was recommended to every patient to improve general conditions, but the final decision was made by patients themselves. This study was approved by the ethics committee of our hospital.

### 2.2. Preoperative Nutritional Therapy

Patients receiving preoperative nutritional therapy were assigned to the NT group, and those on a normal diet were in the non-NT group. For the patients in the NT group, exclusive enteral nutrition (EEN) using a polymeric formula (Nutricia, Amsterdam, Netherlands) which was infused continuously through a nasogastric tube was the first choice. Any other foods and drinks except water were forbidden. If the goal calorie intake (20–25 kcal/kg body weight per day) could not be achieved by enteral nutrition (EN) alone, EN combined with parenteral nutrition (PN) was implemented. Patients with complete food intolerance such as complete intestinal occlusion were given total parenteral nutrition (TPN).

### 2.3. Other Preoperative Managements

Preoperative managements except preoperative nutritional therapy were similar in the two groups. Weaning of steroids was achieved before surgery. Any other drugs, like sulfasalazine, azathioprine, and anti-TNF-*α* therapy, and smoking were stopped as soon as the patient was hospitalized. If an abscess/fistula was present, a closed double-lumen irrigation-suction tube was performed depending on the location and size/type of the abscess/fistula. Antibiotics were used only when there were evidences for infections.

### 2.4. Surgical Procedure

Open surgical resection was performed using midline incision for all patients by the same surgeon team. After vascular and bowel division, a stapled side-to-side anastomosis was performed using the linear cutter 50 mm and linear stapler 75 mm.

Indications for a temporary diverting stoma were presence of abscess irrespective of drainage before surgery and/or severe intestinal edema and/or ≥2 anastomosis performed and/or complex fistula requiring large intestinal resection. Faecal diversion was performed using end stoma. Delayed anastomosis was performed 3–6 months later when the general condition of the patient had improved.

### 2.5. Outcome Definitions

The primary outcome was anastomotic leakage within 30 days of surgery. Secondary outcome was temporary diverting stoma intentionally performed at the time of surgery and not for postoperative anastomotic leakage.

### 2.6. Statistics

Continuous variables were expressed as mean ± standard deviation, and categorical variables were expressed as frequencies and percentages. Unpaired* t*-test was used for two groups' continuous data comparison, and Chi-square test or Fisher's exact test was performed for two groups' categorical data comparison. Factors for anastomotic leakage were evaluated by use of univariate analysis and multivariate logistic regression analysis. All variables tested in the univariate analysis with a *P* value of <0.20 were included in a multivariate logistic regression analysis. All analyses were performed using SPSS, Version 17.0 (SPSS Inc., Chicago, IL, USA). *P* values <0.05 were considered significant.

## 3. Results

### 3.1. Baseline Characteristics

One hundred and fourteen patients were enrolled. Of these 114 patients, six patients underwent a secondary surgery and one patient underwent fourth surgeries, so the total number of surgeries was 123. Among these 123 surgeries, 66 surgeries were in the non-NT group while 57 surgeries were included in the NT group. NT and non-NT groups had comparable patients characteristics. Most patients (62.1% in non-NT group and 64.9% in NT group) underwent surgeries due to penetrating CD (fistula or abscess) (Table 1).

### 3.2. Preoperative Managements and Operative Procedures

Table 2 reported the preoperative managements. Mean preoperative management time was 11.3 ± 4.7 days in non-NT group and 22.7 ± 8.2 days (*P* < 0.0001) in the NT group. In NT group, 48 patients (84.2%) were treated with preoperative EEN. Three patients received TPN due to complete intestinal occlusion, and six patients received combined EN and PN. When comparing other preoperative managements, no difference was noted. Drainage was performed for most patients with penetrating CD. Operative procedures were also listed in Table 2. Most patients underwent small-bowel resection or partial colectomy.

### 3.3. The Serum Albumin and CRP Levels before and after Preoperative Management

**Figure 1:**
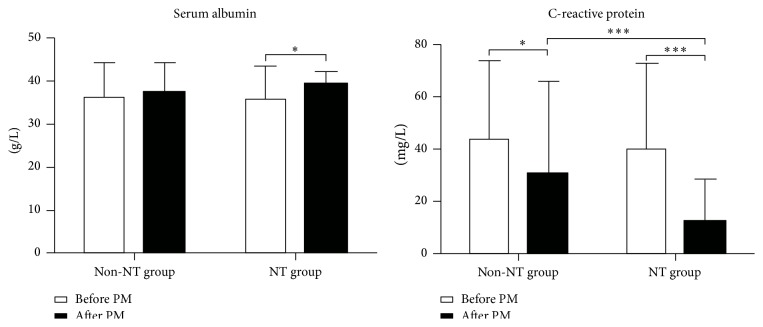
Serum albumin and C-reactive protein levels before and after preoperative managements. PM, preoperative managements; ^*∗*^ *P* < 0.05; ^*∗∗∗*^ *P* < 0.001.

After preoperative managements, CRP and albumin levels improved significantly in NT group, while only CRP decreased remarkably in non-NT group. Patients in NT group had a significantly lower level of CRP compared with patients in non-NT group (Figure 1).

### 3.4. Anastomotic Leakage and Temporary Diverting Stoma

Anastomotic leakage was investigated in surgeries without a stoma. In total, 83 patients underwent anastomosis (39 patients in Non-NT group and 44 patients in NT group). Eight patients suffered anastomotic leakage (Table 3). Patients in NT group had a lower incidence of anastomotic leakage (2.3% versus 17.9%, *P* = 0.023). The rate of reoperation due to anastomotic leakage was similar between these two groups (Table 4).

Temporary diverting stomas were performed in 40 patients in total. The rate of temporary stoma was significantly lower in NT group (13, 22.8% versus 27, 40.9%, *P* = 0.036). In non-NT group, three patients had a temporary stoma because of both severe intestinal edema and complex fistula requiring large intestinal resection, one patient had the stoma due to both the presence of abscess irrespective of drainage before surgery and ≥2 anastomosis performed, and one stoma was performed due to both complex fistula requiring large intestinal resection and ≥2 anastomosis performed. In the NT group, indications for one temporary stoma were both complex fistula requiring large intestinal resection and ≥2 anastomosis performed. As showed in Table 5, the number of temporary stomas due to the presence of abscess irrespective of drainage before surgery, severe intestinal edema, or complex fistula requiring large intestinal resection was lower in the NT group. However, only the difference in severe intestinal edema was significant.

### 3.5. Risk Factors for Anastomotic Leakage

Nine factors were tested to identify predictive factors of anastomotic leakage: age at operation, serum albumin ≤35 g/L, serum CRP > 10 mg/L, preoperative nutritional therapy, penetrating behavior as the indication for resection, preoperative corticosteroids, intraoperative abscess, being active smoker, and preoperative azathioprine or* Tripterygium wilfordii* Hook. F. (a Chinese herbal drug with immunosuppressive effect). In univariate analysis, preoperative nutritional therapy (*P* = 0.023) was associated with the risk of anastomotic leakage (Table 6). The multivariate analysis identified serum albumin of the day before surgery ≤35 g/L (*P* = 0.030) and preoperative nutritional therapy (*P* = 0.023) were factors which independently affected the risk of anastomotic leakage (Table 6).

## 4. Discussion

In CD, due to the risk of anastomotic leakage after intestinal resection, a temporary stoma is often needed, especially for high-risk patients. However, most CD patients requiring surgeries suffer at least one suspected risk factor of postoperative complications, which means improving patients' general condition through preoperative managements is necessary to reduce the risk of anastomotic leakage and then decrease the formation of a temporary stoma. Our study suggested that patients who received preoperative nutrition had a lower risk for a temporary stoma after bowel resections and a lower rate of anastomotic leakage in surgeries without stoma compared with those on a normal diet. Also, serum albumin of the day before surgery ≤35 g/L was identified to be one risk factor for anastomotic leakage.

The rate of anastomotic leakage in our study was decreased from 17.9% to 2.3% through preoperative nutrition. This 2.3% rate in NT group compared favorably with previous studies, ranging from 1.8% to 19% [4, 5, 11, 13, 14]. In a recent study, Li et al. found that 3-month EEN before surgery reduced the rate of anastomotic leakage from 11.8% to 1.8% in patients with fistulizing CD, which is consistent with our results [11]. The frequency of temporary diverting stoma formation was decreased from 40.9% to 22.8% in our study, which also compared favorably with previous two studies. In these studies, Goyer et al. reported that temporary stoma was performed in 39% of patients with complex CD (fistula, abscess, and recurrent disease after ileocolonic resection), and Melton et al. found that the rate was 51% in CD patients with ileosigmoid fistula [6, 7]. However, when comparing with a previous study using the similar preoperative managements, our rate of temporary stoma was higher (7.7% versus 22.8%) [12]. This difference may be due to the different indications for a stoma adopted by these two surgeon teams during operations.

Our results suggested that preoperative nutritional therapy improved operative outcomes in terms of temporary diverting stoma and anastomotic leakage. But the exact mechanics remain unclear. Poor nutritional status and low preoperative albumin levels have been demonstrated to be associated with higher rates of anastomotic complications [3, 5]. Preoperative nutrition can improve nutritional status in CD patients requiring surgery. Li et al. found that preoperative nutrition increased the level of serum albumin in patients with fistulizing CD, and we found that patients in NT group had a higher level of albumin. Improvement of nutritional status alone in preoperative situation may be insufficient to explain the benefits of preoperative nutrition in CD. Accumulating studies demonstrated that nutritional therapy in CD could reduce intestinal and mesenteric fat inflammation by reducing proinflammatory cytokine expression. This anti-inflammation effect may be able to improve the capacity of wound healing [15–17]. We also observed, after preoperative nutrition, that patients in the NT group had a significantly lower level of CRP, a useful marker of inflammation. As showed in Table 3, stoma performed due to severe intestinal edema was significantly reduced in NT group. There were also fewer patients who need stoma due to complex fistula requiring large intestinal resection in NT group. This may be the result of the combination of the improvement of nutritional status, correction of hypoalbuminaemia, and the anti-inflammatory effect of preoperative nutritional therapy in CD. The similar effect of nutritional therapy was reported in two recent studies. One showed that 3-month EEN could lead to closure of enterocutaneous fistula in 30/48 CD patients, and the other one reported that 3-month EEN relieved inflammatory bowel stricture, reflecting a significant increase of luminal cross-sectional area by relieving bowel wall edema in patients with CD [18, 19].

In the present study, serum albumin of the day before surgery ≤35 g/L was determined as a mainly risk factor of anastomotic leakage, which has been confirmed by previous studies. This can partly explain why preoperative nutrition could improve outcomes of surgery as serum albumin increased significantly in NT group after therapy. Some studies have reported that the penetrating behavior was more aggressive than the others and was an independent risk factor of postoperative intra-abdominal septic complications and postoperative recurrence [3, 5, 20–22]. However, our results did not find this relativity. This could be due to the small size (123 surgeries) and heterogeneity (including both ileal resection and colonic resection and both primary resection and multiple resection) of our population.

There are some limitations of this study. Firstly, due to the small, heterogeneous patient population, the results may be overestimated. Secondly, in the present study we did not investigate the outcomes of a long-term follow-up which may be useful. Finally, different from some previous studies, in which stoma was considered on patients with two or more risk factors prior to surgery, our decisions on temporary stoma were all made intraoperatively, and some indications for a stoma were subjective like “severe intestinal edema” [4]. This may limit the generalization of our results.

## 5. Conclusion

In CD patients requiring intestinal resection, preoperative nutritional therapy could reduce the risk of anastomotic leakage while decreasing the frequency of temporary diverting stoma formation. Serum albumin of the day before surgery ≤35 g/L was an independent risk factor for anastomotic leakage.

## Figures and Tables

**Table 1:** Patients characteristics.

Baseline features	Non-NT group (*n* = 66)	NT group (*n* = 57)	*P* value
	*n* (%)	*n* (%)	
Age (years)	33.3 ± 10.1	32.6 ± 10.8	0.734
Gender (male/female)	53/13 (80.3/19.7)	39/18 (68.4/31.6)	0.149
Body mass index (kg/m^2^)	17.8 ± 3.4	18.4 ± 4.6	0.409
Active smoker	23 (34.8)	25 (43.9)	0.356
Duration of CD (years)	8.1 ± 6.1	8.3 ± 7.4	0.870
Disease location			0.644
L1 (ileum)	11 (16.7)	13 (22.8)	
L2 (colonic)	9 (13.6)	5 (8.8)	
L3 (ileocolonic)	42 (63.6)	34 (59.6)	
L4 (upper GI) + L1	2 (3.0)	1 (1.8)	
L4 + L3	2 (3.0)	4 (7.0)	
Behavior			0.74
B2 (stricturing)	25 (37.9)	19 (33.3)	
B3 (fistulizing)	41 (62.1)	38 (66.7)	
Previous intestinal resection	6 (9.1)	3 (5.3)	0.502
Preoperative corticosteroids	12 (18.2)	9 (15.8)	0.812
Preoperative azathioprine or TWP	31 (47.0)	26 (45.6)	1.0
Serum CRP before preoperative management	43.7 ± 30.2 mg/L	40.2 ± 32.7 mg/L	0.539
Serum albumin before preoperative management	36.1 ± 8.1 g/L	35.8 ± 7.7 g/L	0.834

*n*, number of surgeries; TWP, *Tripterygium  wilfordii* Hook. F.

**Table 2:** Preoperative management and surgical procedure.

Management	Non-NT group (*n* = 66)	NT group (*n* = 57)	*P* value
	*n* (%)	*n* (%)	
Preoperative management time (days)	11.3 ± 4.7	22.7 ± 8.2	<0.0001
Nutritional therapy			
EEN	—	48 (84.2%)	
EN and PN	—	6 (10.5%)	
TPN	—	3 (5.3%)	
Drainage	41	35	1.00
Cessation of drugs			0.67
Steroids	12 (18.2)	9 (15.8)	
Azathioprine or TWF	31 (47.0)	26 (45.6)	
Anti-TNF-*α*	1 (1.5)	3 (5.3)	
Sulfasalazine or mesalazine	16 (24.2)	12 (21.1)	
Antibiotics	13 (19.7)	9 (15.8)	0.64
Surgical procedure			0.63
Small-bowel resection	23	24	
Ileocolic resection	13	11	
Ileocecal resection	7	9	
Partial colectomy	18	10	
Other procedures	5	3	

*n*, number of surgeries; TWP, *Tripterygium  wilfordii* Hook. F.

**Table 3:** Details of patients with anastomotic leakage.

Patient number	1	2	3	4	5	6	7	8
Age (years)	26	24	31	29	19	46	41	28
Gender (male/female)	M	F	M	M	F	F	M	M
Active smoker	No	No	No	No	Yes	Yes	No	Yes
Duration of CD (years)	3.3	4	1.8	9	1.3	11	6	9.3
Disease location	L3	L3	L2	L3	L3	L3	L2	L3
Behavior	B3	B3	B3	B3	B2	B3	B3	B3
Preoperative corticosteroids	Yes	No	No	No	No	No	Yes	No
Preoperative azathioprine or TWP	No	Yes	No	No	Yes	Yes	No	No
Serum CRP before/after preoperative management	37.3/15.4	21.3/9.4	50.4/21.7	42.0/5.5	96.1/37.8	40.5/60.7	53.2/46.6	36.0/19.0
Serum albumin before/after preoperative management	36.2/38.7	28.9/31.1	34.7/36.6	36.9/40.2	30.0/28.6	37.2/34.4	35.0/35.5	33.1/31.9
Nutritional therapy	Yes	No	No	No	No	No	No	No
Drainage	Yes	Yes	Yes	Yes	No	Yes	Yes	Yes
Antibiotics	No	No	No	No	Yes	No	No	No
Surgical procedure	Ileocolic resection	Ileocolic resection	Partial colectomy	Ileocolic resection	Ileocolic resection	Ileocolic resection	Partial colectomy	Ileocolic resection

**Table 4:** Anastomotic complications.

Complications	Non-NT group (*n* = 39)	NT group (*n* = 44)	*P* value
	*n* (%)	*n* (%)	
Anastomotic leakage	7 (17.9)	1 (2.3)	0.023
Reoperation due to anastomotic leakage	3 (7.7)	1 (2.3)	0.338

*n*, number of surgeries without stoma.

**Table 5:** Temporary stoma.

Indications	Non-NT group (*n* = 66)	NT group (*n* = 57)	*P* value
	*n* (%)	*n* (%)	
Presence of abscess irrespective of drainage before surgery	4 (6.1)	1 (1.8)	0.372
Severe intestinal edema	8 (12.1)	1 (1.8)	0.037
≥2 anastomosis performed	6 (9.1)	4 (7.0)	0.751
Complex fistula requiring a large intestinal resection	14 (21.2)	8 (14.0)	0.351
Total	27 (40.9)	13 (22.8)	0.036

*n*, number of surgeries.

**Table 6:** Univariate analysis and multivariate analysis for predictive factors of anastomotic leakage.

Variables/categories	Anastomotic leakage		Univariate analysis *P* value	Multivariate Analysis *P* value
	Absent (*n* = 75)	Present (*n* = 8)		
Age (years)			1	
≤40	51	6		
>40	24	2		
Indications for resection			0.130	0.382
Penetrating type	41	7		
Other types	34	1		
Serum albumin of the day before surgery			0.051	0.030
≤35 g/L	13	4		
>35 g/L	62	4		
Serum CRP of the day before surgery			0.142	0.79
≤10 mg/L	49	2		
>10 mg/L	26	6		
Preoperative corticosteroids			0.605	
Yes	11	2		
No	64	6		
Preoperative azathioprine or TWP			1	
Yes	33	3		
No	42	5		
Intraoperative abscess			0.129	0.131
Yes	11	3		
No	64	5		
Active smoker			1	
Yes	25	3		
No	50	5		
Colonic disease			0.286	
Yes	9	2		
No	66	6		
Preoperative drainage			0.675	
Yes	54	7		
No	21	1		
Preoperative nutritional therapy			0.023	0.023
Yes	43	1		
No	32	7		

*n*, number of surgeries; TWP, *Tripterygium  wilfordii* Hook. F.
